# Supplementary material for: Soil temperatures and active carbon components as key drivers of C stock dynamics between two different stand ages of Larix principis-rupprechtii plantation
Source: PeerJ. 2020 Jan 21;8:e8384. doi: 10.7717/peerj.8384 (PMC6979437; doi:10.7717/peerj.8384)
Supplement: Supplemental Information 2 [file peerj-08-8384-s002.doc]

| **PLFA biomarkers for microbial group** | |
| --- | --- |
| **Microbial group** | **PLFA biomarkers** |
|  |  |
| **Bacteria** | 14:00 |
|  | 15:00 |
|  | 16:00 |
|  | 16:1w5c |
|  | 17:00 |
|  | 18:00 |
|  | 20:00 |
| **Gram-negative bacteria** | 14:1w5c |
|  | 15:1w6c |
|  | 16:1w7c |
|  | 16:1w7t |
|  | 16:1w5c |
|  | 16:1w9c |
|  | 17:1w8c |
|  | 18:1w9c |
|  | 18:1w7c |
|  | 18:1w7t |
|  | cy17:0 |
|  | cy19:0 |
| **Gram-positive bacterium** | i13:0 |
|  | a13:0 |
|  | i14:0 |
|  | i15:0 |
|  | a15:0 |
|  | i16:0 |
|  | i17:0 |
|  | a17:0 |
| **Fungi** | 10Me 18:0 |
|  | 10Me 16:0 |
|  | 10Me17:0 |
|  | 16Me16:0 |
| **Fungus** | 18:2w6c |
|  | 18:3w6c |
|  | 18:3w3c |
|  | 18:1w9c |
| **Protozoon** | 20:2w6 |
|  | 20:3w6 |
|  | 20:4w6 |
| **MethanogensI** | 16：1w8c |
|  | 16：1w6c |
| **Methanogens** | 18：1w8c |
|  | 18：1w8t |
|  | 18：1w6c |
| **Aerobic bacteria** | i15:0 |
|  | a15:0 |
|  | 15:00 |
|  | i16:0 |
|  | 16:1w9 |
|  | 16:1w7t |
|  | i17:0 |
|  | a17:0 |
|  | 17:00 |
| **Anaerobic bacteria** | 18:1w7c |
|  |  |
